# Understanding health systems, health economies and globalization: the need for social science perspectives

**DOI:** 10.1186/1744-8603-8-30

**Published:** 2012-08-31

**Authors:** Susan F Murray, Ramila Bisht, Rama Baru, Emma Pitchforth

**Affiliations:** 1 King’s College London, 57 Waterloo Road, London, SE1 8WA, UK; 2 Centre of Social Medicine and Community Health, Jawaharlal Nehru University, New Mehrauli Road, New Delhi, 110067, India; 3 RAND Europe, Westbrook Centre, Milton Road, Cambridge, CB4 1YG, UK

## Abstract

The complex relationship between globalization and health calls for research from many disciplinary and methodological perspectives. This editorial gives an overview of the content trajectory of the interdisciplinary journal ‘Globalization and Health’ over the first six years of production, 2005 to 2010. The findings show that bio-medical and population health perspectives have been dominant but that social science perspectives have become more evident in recent years. The types of paper published have also changed, with a growing proportion of empirical studies. A special issue on ‘Health systems, health economies and globalization: social science perspectives’ is introduced, a collection of contributions written from the vantage points of economics, political science, psychology, sociology, business studies, social policy and research policy. The papers concern a range of issues pertaining to the globalization of healthcare markets and governance and regulation issues. They highlight the important contribution that can be made by the social sciences to this field, and also the practical and methodological challenges implicit in the study of globalization and health.

## Background

The peer-reviewed, online open-access journal *Globalization and Health* was established in [2005] with the aim of providing an international forum for high quality original research, knowledge sharing and debate on the topic of globalization and its effects on health, both positive and negative. Within its stated scope the journal recognises the complexity and breadth of topics and the range of disciplinary perspectives required to understand the relationship between globalization and health. In this editorial and special issue we pay attention to the particular contribution of social science. Social scientists, including economists, political scientists and sociologists, have undoubtedly been key contributors to the discussions and theorising about globalization processes since they began to use the term in the [1960]s, long its current widespread use. That theoretical armoury is combined with research approaches that lend themselves well to exploration of the micro, meso and macro forces that confront health systems in the globalizing world, and one would expect to see prominent participation of these disciplines in current published research in this field. The review paper by Bisht et al. [1] published in this special issue examines the broader ‘state of the art’ in this regard using the case of research on India, and gives suggestions for future ways forward. [1] We also undertook a mapping of this journal’s own content in order to track trends, emphases, commonalities and differences in the work published over the first six full years of its operation ( [2005,2010]) and to locate the place of social science within its content so far. Ninety four papers were reviewed for topic, author’s institution, disciplinary perspective, geographical focus, methodology and funding. Topics were then grouped into more general themes.

## ‘Globalization and Health’: the first six years

The topic areas of HIV/AIDS and globalization and food, diet and obesity have been consistent themes throughout the early years of the journal, as have access to medicines and issues concerning TRIPS and trade agreements. Non-communicable and chronic diseases have seen a growth in the later years, particularly in [2009] and [2010] with the special issue dedicated to ‘Africa’s chronic disease burden: local and global perspectives’, reflecting the greater attention being given to non-communicable diseases (NCDs) at a global level. Some topics have received concentrated but one-off attention such as a mini-series of papers relating to globalization and social determinants of health published in [2007][2,4] to coincide with the Interim Statement of the Commission on Social Determinants of Health.

Most papers published in the initial period of publication focus on how economic development and trade may impact on proximal determinants of health, confirming the need for a new journal with the scope for such work. A number of papers also focus on what Huynen et al. [5] refer to as institutional responses, looking at global governance and policy issues (e.g. [6-9]). Less attention has been given to the role of global communication, global mobility and cross-cultural interaction and, despite its currency, very little to the impact of global environmental changes.

There has been a notable change in the type of study published in this journal over time. All but four of the papers published in 2005 and 2006 were non-empirical in content. These included editorials, debates, reviews without explicitly reported methodology, and conceptual work. In contrast, in years 2008-2010 these sorts of contributions comprised only around half of papers published each year. In total, out of 94 papers across the six years 10 presented primary research, 15 secondary analysis and 7 structured or systematic literature reviews. Methodological approaches used in studies began to represent the diversity that is needed in this field, ranging from macro-level quantitative research such as a statistical analysis of effects of globalization on health [10] analysis of the dynamics of global antiretroviral medicine markets [11] to micro level, inductive studies aimed at explaining how processes of globalization are experienced [12] such as that of Read et al. ( [2009]) exploring local suffering within global discourses on mental health and human rights [13]. In general more papers (n = 53) refer to a ‘global’ context or to developing countries in general, and fewer give focus to individual countries or specific world regions (n = 32). In 2009 and 2010 there was an increase in the number of papers focusing on specific countries and this may reflect the increasing number of empirical studies published during this time.

Sources of research funding were poorly reported but where information was available there was a fairly equal spread between public (n = 15) and private sources (n = 19). Public funding sources included government departments such as the Department for International Development, UK and national research councils. Private sources included pharmaceutical companies, not-for-profit organisations, private foundations and university departments in US universities. For the vast majority of papers (n = 87) the first author was affiliated to an institution in a high income country, despite the publisher’s policy to waive the article processing fee for authors from low-income countries and long standing calls to ensure research from developing countries is represented in international health literature Langer et al. [2004][14].

The majority of papers each year, and 54 out of 94 in total, have lead authors with a bio-medical or population health perspective (including epidemiology and public health) but in more recent years a greater proportion of social science papers have been published rising to 9 out of 23 in 2010. No one social science discipline dominated and there have been contributions from economics (incorporating health economics), psychology, political science and international studies, demography, development studies, management and business studies, human geography, social policy, sociology and anthropology.

## The special issue on social science approaches

This brief review of papers points to several important gaps in the initial coverage of *Globalization and Health,* some of which mirror challenges facing the wider international publishing field*.* One important conclusion is that, despite its importance to the field, social science has been somewhat under-represented in the journal to date. This situation begins to be addressed with publication of this special issue, a selection of peer reviewed papers from those presented at a research sympo-sium organised by King’s College London, Jawaharlal Nehru University and LSE Health, London School of Economics and Political Science (LSE) on this theme in July 2010. The international meeting was held at the LSE and supported by the UK Economic and Social Research Council (ESRC). The special issue brings together review articles, reports of original research studies and concept papers that reflect current developments within a range of social science disciplines contributing to this area of enquiry. The aim of the collection is to highlight challenges and innovative approaches and to inform future research agendas. The full collection can accessed at http://www.globalizationandhealth.com/series/social_science_perspectives

The special issue draws together empirical and non-empirical studies from different disciplines with first authors from economics [15-18], political science [19], psychology [1], sociology [20], business studies [21], social policy [22]. It also includes a perspective from public health that takes up the case for greater funding of social science research [23].

Bisht et al. ( [2012])’s systematic scoping exercise directly investigates the current social science contribution to an understanding of globalization and healthcare using the lens of India. Using an extensive multistage search process across electronic databases, journal indexes and books, the paper maps social science contributions in seven thematic areas: transnational movement of healthcare workforce; transnational consumption of services; production, consumption and trade in specific healthcare related commodities; transnational diffusion of ideas and knowledge; new global governance issues and structures; transnational delivery of services; and transnational movement of capital [1]. The review demonstrates that there is a recent and expanding literature with some important empirical studies despite inherent difficulties such as obtaining global comparative data and in accessing commercially sensitive information.

The remaining articles in this issue fall into two broad thematic areas: Globalization of healthcare markets and Governance and regulation issues.

### Globalization of healthcare markets

Early content of this journal gave relatively little attention to transnational trade and delivery of health services, and the current transnational movement of health care providers, consumers or capital. A number of the papers in this special issue address this deficit, with research relating directly to the globalization of health care markets.

Three of the contributions explore the potential for bi-lateral agreements between India and other countries. Chanda (2011) considers the opportunity for and constraints to India-European Union (EU) relations in health services against a backdrop of the India-EU Trade and Investment Agreement (TIA) currently under negotiation [18]. The paper draws on interviews with management and practitioners from a variety of healthcare establishments in four Indian cities as well as official representatives from the Indian and EU countries. The paper argues that whilst there are evident opportunities for trade, for example, in the case of e-health services and medical value travel, significant steps would be required before realising these. Not least, concerns about the commercialization of health services would have to be overcome by EU partners who are more comfortable with and confident of public sector healthcare delivery. Two further papers by Martinez Alvarez and colleagues [16,17] explore in more depth the potential for bi-lateral agreements between India and the UK for medical tourism and for telemedicine. Using a similar approach -structured interviews with stakeholders in the UK and India - these papers highlight the many concerns and a degree of scepticism that remain around bi-lateral agreements.

Lethbridge (2011) is an example of the value of ‘cross-fertilization’ of ideas to illuminate health questions [21]. In her qualitative study of the actions and motives of five multinational companies engaged in provision and management within public health systems she draws on Porter’s Five Forces theory of company expansion, a business strategy framework not often applied to studies in health policy. Pocock and Phua (2011) draws on experience of Thailand, Singapore and Malaysia in order to propose a conceptual framework to understand medical tourism and its policy implications for health systems [22]. The framework identifies five key components for consideration and future empirical analysis: governance; regulation; delivery; financing and human resources. The authors highlight that whilst medical tourism can bring economic benefits for countries this may come at the expense of access and use of health services by the local population and that policies in these five areas must be adequately addressed at a national level.

Sarojini et al. ( [2011]) consider the specific issue of the globalization of ‘birth markets’ and the equity and ethical implications of growth of Assisted Reproductive Technologies (ART) in India [24]. The paper draws on exploratory qualitative research undertaken by SAMA Resource Group for Women and Health, a Delhi-based resource group working on gender, health and rights. Their work captures what Brown & Labonte (2011) have referred to as the dialectical features associated with globalization [12]. The paper maps the growth and features of the fertility industry in India including actors, costs, marketing and regulation and sets this against the concern about exploitation and the failure to ensure wellbeing, rights and security for the women involved. In conclusion it raises the important question, “how can we ensure that the crossing of geographic and ‘biological’ boundaries does not become a crossing of ethical boundaries?”.

### Governance and regulation

Four papers consider issues of governance and regulation, two of these within the sphere of pharmaceuticals. Iriart *et al.* (2011) consider the challenges for health sector regulation in Latin America following the liberalisation of markets and expansion of neo-liberal ideas [20]. Drawing on primary and secondary sources, the authors use the concepts of biomedicalization and biopedagogy to show how the pharmaceutical industry has developed strategies to increase their share of the health market. The authors argue that regulatory agencies in both developed and developing countries lack capacity to keep pace with and regulate data gathering and communication tools that the multinational corporations create to reach their populations. Mackintosh et al. ( [2011]) consider the potential role of non-governmental organisations (NGOs) in pharmaceutical market regulation, given the recognised lack of adequate regulation in countries such as India and Tanzania [15]. Based on data from interviews with trading NGOs and social enterprises operating in Europe, India and Tanzania, the authors apply a socio-legal and economic perspective to assess the activities of these enterprises in essential medicines wholesaling. Their findings suggest that social enterprise wholesaling can improve access to medicines in the absence of effective governmental activity but that it should not replace state action.

Salter & Faulkner (2011)’s concern is the governance of global life science and biomedical innovations with particular reference to the ‘Rising Powers’ such as India and China [19]. They draw on contributions from across political science, political economy, sociology of technology, innovation studies and science and technology studies. Following a thorough review of existing conceptual approaches, Salter and Faulkner conclude that an approach is required that “enables innovation and governance to be seen as ‘co-producing’ each other in a multi-level, global ecology of innovation, taking account of the particular, differing characteristics of different emerging scientific fields and technologies”.

The final paper comes from a public health commentator who argues strongly for greater input and recognition for social science in research policies. McCarthy (2011) considers how global knowledge transfer has long been an important driver of cultural and economic development [23]. Reviewing current policies of the European Commission that promote science for innovation within European member states and the international transfer of people and ideas, he concludes that there is too much attention on biomedical innovation at the expense of the need for innovation in health and social systems and for increased participation of civil society organisations that will be required to meet the challenges of globalization on health.

## Concluding comment: the practical and methodological challenges

This editorial began by highlighting the dominance of ‘population health science’ perspectives and the relative lack of social science perspectives in papers published in this journal *Globalization and Health* during its early years. The papers we have introduced in this special issue make a contribution to redressing that balance and in doing so they demonstrate the valuable contribution that social sciences can make in questioning the taken-for‐granted assumptions of prevailing ideology, in developing illuminating theory, and in testing and modifying existing theory in the light of empirical situated data. The range of disciplinary perspectives captured in the special issue is broad and reflective of the range of disciplines that may contribute to the understanding of the complex relations between globalization and health. Where there is less diversity is in the research methods that were employed and this highlights a persistent challenge for researchers concerned with the study of globalization and health. An understanding of the relationships between globalization and health requires perspective on macro, meso and micro levels, and innovative methodological approaches to enable this. Bisht et al. [1] highlight, for example, that the extended case study method as one approach seldom used in the health sphere that may have particular purchase through its ability to move between the micro to macro [25]. Similarly, Brown and Labonte have recently highlighted the potential of qualitative methods in particular in understanding how the “intersects of globalization are manifested in particular locations” [12]. The journal invites further contributions to this discussion and warmly encourages submissions that explore and employ new methodology that will help to extend our understanding of the field and of the accompanying processes of social change.

## Competing interests

EP is a co-Editor-in-Chief of the journal Globalization and Health (2008 onwards). SFM is a member of the Globalization and Health Editorial Board (2011 onwards).

## Authors’ contributions

SFM conceived the idea of the symposium and special issue. SFM, EP and R Bisht reviewed and undertook the data extraction for the papers published in Globalization and Health. EP wrote the first full draft of the paper. SFM, R Bisht and R Baru further revised the paper. All authors have seen and agreed with the final version.

All authors edited the special issue.
